# Change in psychological distress and associated factors among Hong Kong young adults in post-COVID-19 era: a latent transition analysis

**DOI:** 10.1007/s00127-025-02912-5

**Published:** 2025-05-07

**Authors:** Haorui Li, Ted Chun Tat Fong, Yu Cheng Hsu, Wendy Wing Yan So, Tsz Mei Lam, William G. Hayward, Paul Siu Fai Yip

**Affiliations:** 1 Department of Social Work and Social Administration, The University of Hong Kong, Pokfulam, Hong Kong SAR; 2 Centre on Behavioral Health, The University of Hong Kong, Pokfulam, Hong Kong SAR; 3 Research Hub of Population Studies, The University of Hong Kong, Pokfulam, Hong Kong SAR; 4 The Hong Kong Jockey Club Centre for Suicide Research and Prevention, The University of Hong Kong, Pokfulam, Hong Kong SAR; 5 Department of Psychology, Lingnan University, Tuen Mun,, Hong Kong SAR

**Keywords:** Chinese, Post-pandemic, Latent transition analysis, Mental health, Social distress, Young adults

## Abstract

**Purpose:**

The COVID-19 pandemic has brought negative impacts on young adults’ mental health. The present study aimed to examine the transition of psychological distress classes in young adults after the pandemic and the associated factors.

**Methods:**

A total of 577 young adults (mean age = 25.9 years, SD = 4.4) in Hong Kong participated in a longitudinal online survey on mental health in 2022 and 2023. The participants completed the 10-item Chinese Health Questionnaire and self-constructed items on COVID-19 distress, financial distress, and social distress. Latent class analysis was used to classify the participants into latent classes of psychological distress. Latent transition analysis was conducted with measurement invariance to examine the transition amongst the latent classes from 2022 to 2023 and the associations with changes in the stressors.

**Results:**

The data supported three latent classes of psychological distress. A third of the participants belonged to the High-distress class with elevated symptoms and its prevalence decreased from 34.3% to 27.8% over one year. 40.9% and 10.0% of the Moderate-distress and High-distress classes transitioned to the Low-distress class after the pandemic, respectively. Financial distress (OR = 3.14, 95% CI = 1.17–8.41) and social distress (OR = 3.25, 95% CI = 1.70–6.21) was significantly linked to higher odds of transitioning from the Low-distress to High-distress class. Increased social distress was associated with decreased odds (OR = 0.57, 95% CI = 0.39–0.84) of improvement from the High-distress to Moderate-distress class.

**Conclusion:**

The findings suggest an overall reduction in psychological distress among young adults after the pandemic. Increases in financial and social distresses after COVID-19 showed significant effects on worsening psychological distress.

**Supplementary Information:**

The online version contains supplementary material available at 10.1007/s00127-025-02912-5.

## Introduction

The COVID-19 pandemic has resulted in unprecedented global hazards to mental health [1–3]. The World Health Organization announced on May 5th, 2023, that COVID-19 no longer constituted a public health emergency of international concern due to high population-level immunity from infection and vaccination [4]. The pandemic has changed from an acute emergency state to a chronic endemic state [5]. In Hong Kong, the end of the fifth wave of the pandemic in early 2023 marked the beginning of this chronic state. Previous studies have indicated that young people are particularly vulnerable to psychological distress during the pandemic [6–10], as consistently reported by Wong and colleagues that young people in Hong Kong experienced increased psychological distress during the fifth wave of COVID-19 [11]. Despite the emerging evidence on mental health issues among young people in the post-pandemic period [12–13], it remains unclear whether and how psychological distress among young adults has changed in the post-COVID-19 era.

Studies have theorized the relationships between life stressors and mental health specifically in the context of the COVID-19 pandemic [14–16]. The threat of infection was often considered the primary stressor, which indicated the direct impact of COVID-19 on psychological distress [14]. Secondary stressors from pandemic-related life disruptions in the financial and social domains raised concerns about young adults’ mental health [14–16]. Empirical evidence has associated stress attributable to the COVID-19 pandemic with elevated levels of psychological symptoms [14, 17–18]. The pandemic has exacerbated financial insecurity and work challenges, and financial stressors have been linked with poor mental health outcomes [14–17]. Social stressors, such as quarantine and social distancing measures, could result in social withdrawal and isolation, and increased psychological distress [14–16, 18–20]. However, after the high prevalence of infection, the COVID-19 virus may no longer be perceived as a health threat by the public. A lot of government support and financial assistance during the pandemic was no longer available and the restrictive social distancing measures were also lifted. It remains unclear whether the temporal changes in these stressors may have an effect on the changes in mental health.

Previous studies have commonly used variable-centered analytical techniques such as t-tests to describe the changes in psychological distress after the onset of COVID-19 pandemic, but such average trends may obscure individual variations in the temporal changes [10, 21]. To examine the effects of the pandemic, financial and social stressors, and demographic characteristics on young people’s psychological distress, regression analysis and path modelling have been utilized in cross-sectional and longitudinal studies [7–10, 15, 21]. However, these analytic methods are based on the assumption that the effects of the predictors are homogeneous across the study sample, which might not hold in real-life situations.

Person-centered analytic techniques such as latent class analysis (LCA) and its longitudinal variant, latent transition analysis (LTA), can provide fine-grained results on potential heterogeneity in the sample [22–23]. LTA allows estimation of the prevalence and transition among latent classes across time, and examination of factors associated with the transition [24]. Thus, using LTA to analyze the transition of psychological distress after the pandemic would improve our understanding of the changing patterns of psychological distress among young people [25]. This approach will expand upon previous cross-sectional LCA studies to identify latent classes of psychological distress in young adults [26–28], as well as studies tracking the trajectory of change in psychological distress from pre-pandemic to pandemic [29]. It would have clinical relevance to identify individuals who experienced worsening psychological distress and the associated factors. The findings could inform development of policies that are more specific and effective for those identified at risk.

Given the identified research gaps, the present study aims to address the following research questions: (1) What latent classes of psychological distress can be identified among young adults in Hong Kong? (2) How do individuals transition between latent classes from 2022 to 2023 after the COVID-19 pandemic? (3) How do changes in COVID-19 distress, financial distress, and social distress affect the transitions between latent classes of psychological distress?

## Methods

### Study design and procedures

The data used in this study originated from a longitudinal online survey of the OpenUp emotional support project [30]. The survey targeted young people aged 18–35 years old to understand the young generation’s use of the Internet and social services, as well as their psychosocial well-being. A sample of about 2,000 young people was recruited at baseline, with the expectation that between 500 and 1,000 young people would continue to participate in the follow-up survey. Two annual waves were collected in the first quarter of 2022 and the first quarter of 2023, respectively. Since the former covered the peak period of COVID-19 infection in Hong Kong [31] and the restrictions on the pandemic were relaxed in the latter [32], the data could effectively serve the research purpose of this study. Prior to the start of the study, ethical approval had been obtained from the Human Research Ethics Committee of the University of Hong Kong (reference number: EA1709039). The survey was promoted through methods such as sending emails to faculties and students of local universities in Hong Kong, displaying posters at non-governmental organizations, and utilizing websites and social media platforms.

The survey was conducted with the informed consent of the respondents. Participation was voluntary, and all provided information was treated with strict confidentiality. The survey questionnaire took approximately 15 min to complete, and after each round of the survey, the first 2,000 eligible participants received a HK$25 e-voucher as a token of appreciation. After completing the survey, participants were provided with contact information of the 24-hour emotional support hotlines, and online emotional support services for seeking help when experiencing distress.

### Participants

The first wave of the online survey was completed by 1947 participants, and the second wave was completed by 577 participants. The results of the attrition analysis and the descriptive statistics of the final sample are presented in the results section.

### Measures

Demographic information was collected on age, gender (female = 1), education level (post-secondary or above = 1), and a three-category variable on work status coded as two dummy variables (student status = 1 and unemployed = 1) relative to employed (student status = 0 & unemployed = 0) as the reference group. To capture changes in work status over two years, two dummy variables, status change and becoming unemployed, were coded with status remaining the same (status change = 0 & becoming unemployed = 0) as the reference group. The changes from student to work, from work to student, from unemployed to work, and from unemployed to student were coded as status change = 1. The changes from student to unemployed and from work to unemployed were coded as becoming unemployed = 1.

Psychological distress was measured using the adjusted Chinese Health Questionnaire (CHQ10). The original Chinese Health Questionnaire consisted of 12 items developed from the General Health Questionnaire to screen for mild mental disorders [33]. Fong et al. [34] have validated the 10-item version of this scale (CHQ10) in a large sample of Hong Kong youths. The CHQ10 assesses ten psychological distress symptoms (see Supplementary Table 1) experienced by participants in the past two weeks and items were scored on a 4-point Likert scale ranging from 0 (“not at all”) to 3 (“much more than usual”). The total CHQ10 score ranges from 0 to 30 with higher scores indicating worse psychological health. The CHQ10 demonstrated good reliability in the present sample (α = 0.87–0.88).

The COVID-19 distress was measured by the item “*Have you been emotionally distressed by the COVID-19 pandemic?*” on a 5-point Likert scale ranging from 1 (“not at all”) to 5 (“extremely”). Financial distress was measured by the item “*Did you experience distress in financial circumstance in the past four weeks?*” on a 5-point Likert scale ranging from 1 (“not at all”) to 5 (“extremely”). Social distress was measured by three self-constructed items on the experienced distress in relations with colleagues, friends, classmates, with spouse or partner, and with family members over the past 4 weeks. The items were rated on a 5-point Likert scale ranging from 1 (“not at all”) to 5 (“extremely”). The three-item self-constructed scale showed acceptable composite reliability (α = 0.68–0.75) and the average score was calculated to represent the overall level of social distress, with higher values indicating a higher level of distress. The measurement of stress factors has been adopted in previous studies within the Hong Kong context, demonstrating effectiveness in capturing stressors based on their specific causes [17–18].

### Data analyses

Attrition analysis was performed using chi-squared tests for categorical variables, and independent-sample t-tests for continuous variables. McNemar tests and paired-sample tests were used to test for the changes in categorical variables and continuous variables from 2022 to 2023. Odds ratios (OR) and Cohen *d* were computed to calculate the effect size of categorical variables and continuous variables, respectively.

LTA was subsequently conducted following the framework proposed by Ryoo et al. [24]. In the present study, the CHQ10 items were treated as ordinal categorical variables because half of them showed substantial floor effects, with more than 25% of participants answering the minimum response (0) in both waves. LCA was therefore first conducted on the ordinal CHQ10 items to identify latent classes of psychological distress among young adults in 2022 and 2023 separately using unconditional models (without covariates). The optimal model in each year was selected based on model fit indices, classification quality, and substantive checking. Model fit was evaluated using the Bayesian information criterion (BIC) and the sample-size adjusted BIC (aBIC), with a lower value indicating a better model fit. In case where BIC and aBIC continued to decrease as additional latent classes were added to the model, the elbow point of the model fit (the point at which the decrease in BIC and aBIC began to slow) was taken as the standard for model selection [35]. The bootstrapped likelihood ratio test (BLRT) was used to test the significance of differences in model fit with the addition of one more latent class. Classification precision was assessed by entropy, with a higher value (ideally greater than 0.8) indicating better classification accuracy. Substantive checking of the latent classes of psychological distress was performed with reference to demographic variables (age, gender, education level, student status, and unemployed), COVID-19 distress, financial distress, and social distress under the BCH method [36].

LTA was then conducted to examine the change in prevalence and transition probabilities among the latent classes from 2022 to 2023. Measurement invariance over time was tested by comparing models with and without equal constraints on the item thresholds, to ensure that the meaning of each latent class remained stable over time. The goodness of fit of LTA models was also evaluated by BIC, aBIC, and entropy.

The potential effects of demographic covariates on the transitions in psychological distress were tested in the LTA model. The potential effects of changes in COVID-19 distress, financial distress, and social distress on the transitions in psychological distress classes were tested after controlling for baseline distress and demographic variables in the LTA model. Results were presented as odds ratios with the associated 95% CI. With a maximum missing data rate of 2.1%, sample means are used to impute missing values for continuous variables (i.e., financial distress, social distress), and sample modes are used to impute missing values for categorical variables (i.e., education level). Data cleaning and recoding were conducted using Stata/MP 18.5, and estimations of LCA and LTA models were performed using Mplus 8.11.

## Results

### Sample profiles and descriptive statistics

Attrition analysis (see Supplemental Table S2) found that student status (OR = 1.41, *p* < 0.001) and social distress (*d* = 0.11, *p* < 0.05) showed significant differences between the dropouts (*N* = 1370) and completers (*N* = 577). Those who dropped out of the study were students and showed greater levels of social distress. There was no difference in terms of gender, education level, and unemployment (*p* = 0.14–0.80) and age, the total CHQ10 score, COVID-19 distress, and financial distress (*p* = 0.36–0.79).

As shown in Table 1, the mean age of the final sample (*N* = 577) was 25.9 years (SD = 4.4), and the majority were female (72.6%), had at least post-secondary education (93.7%), and employed (58.8%) in 2022. From 2022 to 2023, the proportion of students in the sample decreased significantly (OR = 0.28, *p* < 0.001), and their total CHQ scores (*d* = 0.15, *p* < 0.001) and COVID-19 distress (*d* = 0.31, *p* < 0.001) decreased significantly, while their financial distress (*d* = 0.23, *p* < 0.001) and social distress (*d* = 0.15, *p* < 0.05) increased significantly. A proportion of 12.8% of the sample experienced a change in work status and 1.4% of the sample became unemployed from 2022 to 2023.

**Table 1:** Sample profiles and descriptive statistics

Variables	2022 M (SD) /%	2023 M (SD) /%
Gender (female)	72.6%	72.6%
Education level (post-secondary or above)	93.7%	96.1%
Student status	38.8%	31.2%
Unemployment	2.4%	1.9%
Age	25.89 (4.42)	26.89 (4.42)
Psychological distress (CHQ10)	11.06 (6.01)	10.17 (5.79)
COVID-19 distress	3.33 (1.03)	2.99 (1.15)
Financial distress	2.51 (1.18)	2.78 (1.18)
Social distress	2.22 (0.84)	2.37 (0.91)
**Variables**	**From 2022 to 2023 (%)**	
Status change	12.8%	
Becoming unemployed	1.4%	

### Latent classes of psychological distress

Table 2 shows the model fit of the unconditional LCA models on psychological distress in 2022 and 2023. At both time points, all of the models showed high entropy (> 0.8) and the BLRTs showed the significance of the differences in model fit with the addition of one more latent class (*p* < 0.001). The BIC and aBIC continued to decrease as more classes were added to the model. As shown by the difference in BIC and aBIC, the BIC and aBIC decreased substantially until the 3-class LCA model, and then decreased slightly as the number of classes increased. The diminishing decrease in BIC and aBIC suggested that the 3-class model was the elbow point of the model fit, and three classes were selected in 2022 and 2023.

**Table 2:** Fit indices of the LCA and LTA models for psychological distress in 2022 and 2023

Model	BIC	Difference in BIC	aBIC	Difference in aBIC	Entropy	BLRT(*p*)
2022: (*N* = 577)						
1-class LCA	14,286	-	14,191	-	-	-
2-class LCA	12,958	1,328	12,764	1,426	0.898	0.000
3-class LCA	12,510	448	12,218	547	0.888	0.000
4-class LCA	12,432	78	12,042	176	0.865	0.000
5-class LCA	12,400	32	11,911	131	0.879	0.000
2023: (*N* = 577)						
1-class LCA	13,863	-	13,768	-	-	-
2-class LCA	12,584	1,279	12,390	1,378	0.893	0.000
3-class LCA	12,200	384	11,908	483	0.878	0.000
4-class LCA	12,178	22	11,787	121	0.855	0.000
5-class LCA	12,164	13	11,676	111	0.852	0.000
2022–2023: (*N* = 577)						
3–3 class LTA without MI	24,538	-	23,941	-	0.900	-
3–3 class LTA with MI	24,092	446	23,781	160	0.882	-

LCA latent class analysis, LTA latent transition analysis, BIC Bayesion information criterion, aBIC adjusted BIC, BLRT(*p*) the *p*-value of Bootstrapped likelihood ratio test, MI measurement invariance

Substantive checking (see Supplemental Table S3) found that in 2022 there were no significant differences in gender, education level, student status, and age among the latent classes (*p* = 0.09–0.75). Unemployment showed a marginally significant difference between the High-distress class and the Low-distress class (*p* = 0.05). In 2023, no significant difference was found among the latent classes in terms of all demographic variables (*p* = 0.20–0.99). COVID-19 distress, financial distress, and social distress showed significant differences among the latent classes in both 2022 and 2023, increasing from the Low-distress class to the High-distress class. These results suggested substantive distinction among the three derived classes in the present sample in 2022 and 2023.

**Fig. 1:**
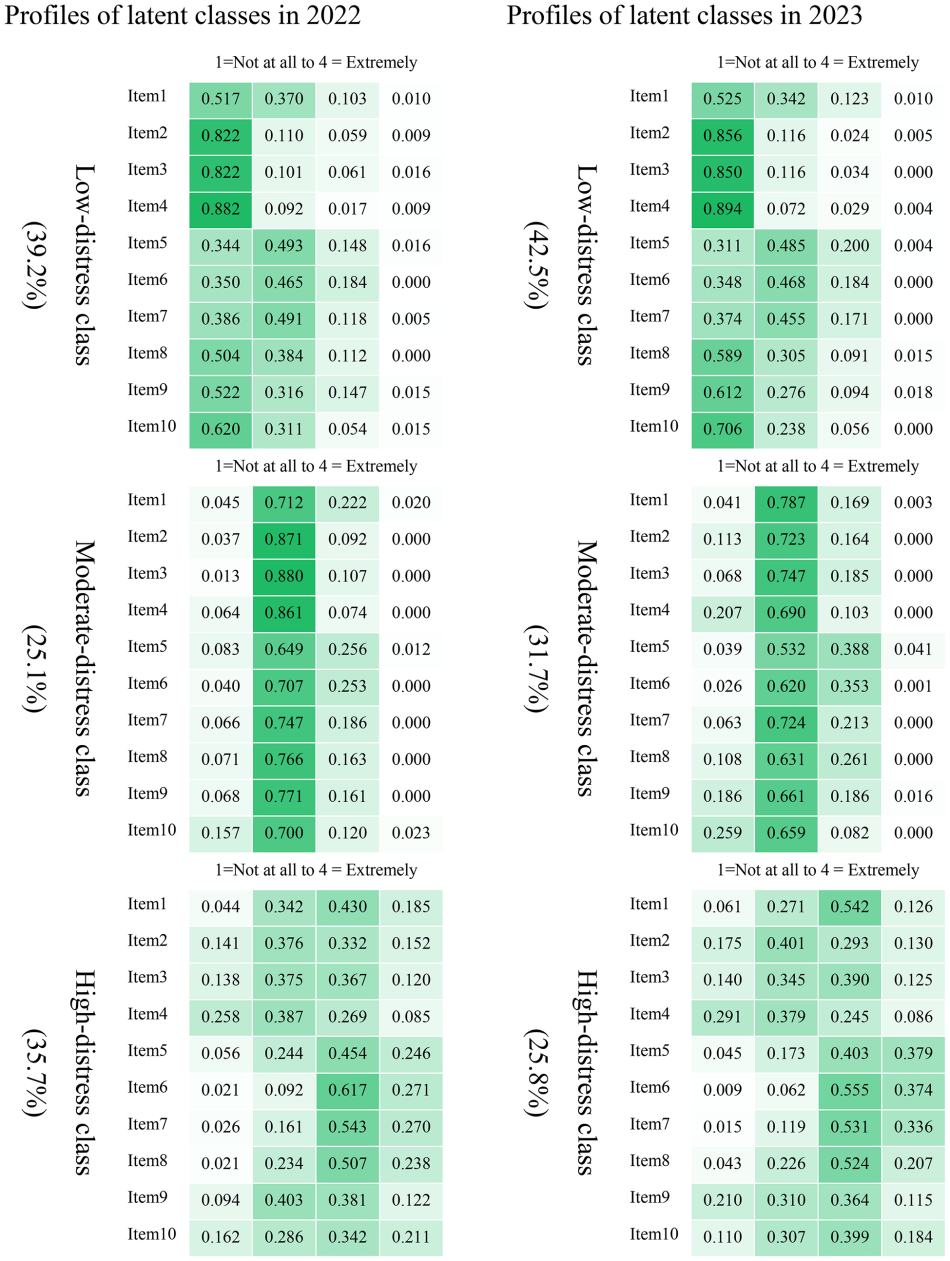
Item probabilities of the three latent classes in 3-class LCA model in 2022 and 2023. *Note.* The darker the color is, the higher the value of item probability

Figure 1 depicts the item probabilities for the 3-class LCA model in 2022 and 2023. In 2022, the sample was divided into three different latent classes: a total of 39.2% were classified in the Low-distress class, with the highest probability of not having most of the symptoms and having only mild symptoms of sleep disturbance, feeling burdensome, and loss of confidence. The Moderate-distress class comprised 25.1% of the sample, exhibiting the highest probability of having all symptoms at a mild level, while the High-distress class included 35.7% of the sample, with the highest probability of having moderate to severe symptoms, especially in terms of sleep problems, feeling burdensome, losing confidence, and feeling nervous. The samples in 2023 showed similar patterns to those in 2022.

### Transition in psychological distress from 2022 to 2023

Measurement invariance was then tested using LTA. After setting the thresholds of all indicators equal across time, the BIC and aBIC decreased and the entropy remained at a high level (> 0.8), supporting the measurement invariance (see Table 2). Table 3 shows the change in prevalence and transition probabilities of the latent classes of psychological distress from 2022 to 2023. The overall prevalence of the High-distress class decreased from 34.3 to 27.8%, while the prevalence of the Moderate-distress class remained stable (28.1–28.5%), and the prevalence of the Low-distress class increased from 37.6 to 43.7%. In terms of changing patterns, the Low-distress and High-distress classes exhibited greater stability compared to the Moderate-distress class, with 76.5%, 58.7%, and 38.9% of individuals in each latent class remaining in the same class membership over time. Many individuals experiencing higher levels of psychological distress in 2022 have shown some recovery; specifically, 40.9% of those in the Moderate-distress class transitioned to the Low-distress class. Additionally, 10% and 31.3% of individuals in the High-distress class moved to the Low-distress and Moderate-distress classes, respectively. Conversely, 18.3% and 5.2% of individuals in the Low-distress class transitioned to the Moderate-distress and High-distress classes, while 20.1% of those in the Moderate-distress class moved to the High-distress class in 2023, indicating a deterioration in mental health status.

**Table 3:** Transition model of psychological distress classes from 2022 to 2023 (*N* = 577)

	Psychological distress class		
	Low-distress	Moderate-distress	High-distress
**Prevalence**			
Baseline(2022)	217 (37.6%)	162 (28.1%)	198 (34.3%)
Follow-up(2023)	252 (43.7%)	165 (28.5%)	160 (27.8%)
**Transition probabilities**			
2022→2023	Low-distress	Moderate-distress	High-distress
Low-distress	*0.765*	0.183	0.052
Moderate-distress	0.409	*0.389*	0.201
High-distress	0.100	0.313	*0.587*

Italicized transition probabilities refer to the same class membership at both times

### Factors associated with transition in psychological distress

Table 4 shows the effects of covariates on transitions between latent classes of psychological distress from 2022 to 2023. None of the demographic variables showed a significant effect on these transition probabilities. After controlling for the demographic variables, baseline values of COVID-19 distress, financial distress, and social distress, it was found that changes in COVID-19 distress did not significantly affect these transition probabilities. However, changes in financial distress were significantly associated with increased odds of transiting from the Low-distress class to the High-distress class (OR = 3.14, 95% CI = 1.17–8.41) among individuals in the Low-distress class in 2022. Similarly, changes in social distress were significantly associated with increased odds of transitioning from the Low-distress class to the High-distress class (OR = 3.25, 95% CI = 1.70–6.21) among individuals in the Low-distress class in 2022, and were also significantly linked to decreased odds of moving from the High-distress class to the Moderate-distress class (OR = 0.57, 95% CI = 0.39–0.84) among individuals in the High-distress class in 2022. Individuals moving from the Low-distress class to the High-distress class were particularly at risk, and further analysis found that this group was mainly young women new to the workplace, with an average age of 25.58 years, predominantly female (91.67%), all with at least post-secondary education, with 75.00% employed in 2022 and 91.67% with stable working status in 2023.

**Table 4:** Odds ratios for covariates predicting transitions between latent classes of psychological distress (*N* = 577)

Predictor	Latent class in 2022	Latent class in 2023 OR (95% CI)			
		Low-distress	Moderate-distress	High-distress	
Change in COVID-19 distress	Low-distress	Ref	0.918 (0.647, 1.303)	1.008 (0.502, 2.026)	
	Moderate-distress	0.899 (0.624, 1.295)	Ref	0.729 (0.443, 1.201)	
	High-distress	0.832 (0.542, 1.279)	0.865 (0.586, 1.277)	Ref	
Change in financial distress	Low-distress	Ref	1.325 (0.863, 2.034)	**3.139 (1.171**,** 8.412)**	
	Moderate-distress	0.905 (0.640, 1.280)	Ref	0.949 (0.532, 1.693)	
	High-distress	0.794 (0.514, 1.226)	1.176 (0.838, 1.650)	Ref	
Change in social distress	Low-distress	Ref	0.782 (0.436, 1.405)	**3.251 (1.702**,** 6.208)**	
	Moderate-distress	0.613 (0.353, 1.065)	Ref	2.053 (0.556, 7.582)	
	High-distress	0.757 (0.437, 1.311)	**0.568 (0.385**,** 0.836)**	Ref	

Bold indicates the significant predictive effects on class transitions. Demographic variables, including age, gender, education level, work status at baseline, and changes in work status, had no significant predictive effects on class transitions of psychological distress across time and were therefore excluded from the table

## Discussion

### Summary of findings

The present study identified three latent classes of psychological distress via LCA among young adults in Hong Kong and examined the temporal change after the COVID-19 pandemic using LTA. Based on model fit indices, classification quality, and the results of substantive checking, the young adults were classified into the Low-distress class, Moderate-distress class, and High-distress class. This is consistent with previous studies on the latent classes of psychological distress [37–38]. The prevalence of the High-distress class decreased from 2022 to 2023. Additionally, both the Moderate-distress and the High-distress classes exhibited higher tendencies to transition to a lower distress class in 2023. Our results suggest a general improvement in mental wellness in young adults in post-COVID-19 era in Hong Kong. This finding is in line with previous studies conducted during the early stages of the pandemic, which indicated that residents could experience worsened mental health during the initial shock of the pandemic, the quarantine measures, followed by a return to approximately pre-pandemic levels of mental health [29, 39].

While an overall decrease in psychological distress among young people was observed, it is important to identify those who transited to higher levels of psychological distress class and the factors associated with their deterioration, and additionally, those who remained at higher levels of psychological distress classes and the barriers hindering their recovery.

Among the factors influencing the transition probabilities of psychological distress, after controlling for age, gender, education level, and work status, only changes in financial and social distress showed significant effects on specific changing patterns, including the transition from the Low-distress class to the High-distress class and from the High-distress class to the Moderate-distress class. These results suggest no differences in patterns of class transition across different demographic groups. This may be because individuals in the same latent class shared similar demographic characteristics at baseline, which may not effectively predict their psychological distress class at follow-up. Additionally, due to the small sample size for the unemployed subgroup (*N* = 11–14), the statistical power may be too low to yield a significant effect on the transition.

To explore the non-significance of changes in COVID-19 distress, a sensitivity analysis was conducted to assess its effects independently while controlling for demographic variables and baseline values of all three stressors. This also found no significant results, indicating that the lack of significant effects from changes in COVID-19 distress was unlikely to be influenced by the other two stressors. Instead, changes in COVID-19 distress may not be a central predictor of the psychological distress transitions. This may be related to a collective reduction in the perception of direct health threats posed by the pandemic after experiencing a widespread infection. Previous studies have indicated that higher awareness of the occurrence and severity of the COVID-19 pandemic was linked with a lower fear, and the COVID-19 pandemic may no longer be perceived as a significant life stressor across all people, with no notable differences among different psychological distress classes [40–41].

Moreover, changes in financial and social distress have shown positive effects on transitioning to or remaining at higher levels of psychological distress class. Specifically, individuals in the Low-distress class were more likely to move to the High-distress class as financial and social distress increased, and change in social distress has negative effects on improvements from the High-distress class to the Moderate-distress class. Previous studies have shown that the COVID-19 pandemic had long-term economic and social consequences, such as a global economic downturn and increased unemployment [42–43]. For young people, being obliged to study or work from home during the COVID-19 pandemic resulted in substantial missed opportunities in terms of educational experiences and social interactions [44]. This put them at a disadvantage in the increased competition for both workers and job seekers, as well as an increased risk of job loss, even after the lockdown policies were lifted [45–46]. Thus, they perceived more financial threats and potential deterioration in relationships with their classmates and colleagues because of the competition, and with their family and partners because of the pressure they were under [47–48]. These changes may thus contribute to the transition to poorer mental health status or remaining high levels of distress [49]. This is particularly the case for people who moved from the Low- to the High-distress class, as they were mainly young women who were new to the workplace and were more sensitive to these financial and social stressors.

Interestingly, the temporal effects of financial and social distress were not found among the Moderate-distress class. This difference may be attributed to the class differences in help-seeking behaviors. Previous studies have found that people in the Low-distress class were less likely to seek help and could suffer more from the effects of increased financial and social distress, especially during the adjustment to returning to school and the worsening of employment conditions after the COVID-19 [50]. Individuals with high levels of psychological distress may depend more on their social network and relationships for support [50]. This could make them more sensitive to changes such as tensions, conflicts, and other negative interactions and amplify the negative effects of increased social distress. People with moderate distress mainly relied on their family, friends, and partner for help, but these supports were not activated during the COVID-19 pandemic restrictions [51]. Following the relaxation of pandemic-related constraint measures, their help-seeking behavior may have normalized, which buffers the impact of financial and social distress among them.

### Implications

The current findings offer insights on future policy design after the pandemic. Despite the lack of a direct effect of the pandemic on young adults’ mental health, the financial and social stressors caused by the pandemic were significantly associated unfavorable transitions in the psychological distress class. The phrase ‘back to normal’ does not necessarily indicate a return to pre-pandemic status, but a new complex landscape with emerging socio-economic challenges such as unfavorable employment conditions, financial pressures, social tensions. In this regard, the financial assistance should be gradually phased out to facilitate smoother transition into the ‘post-COVID-19 era’. Additionally, students need to have more time to catch up with the curriculum and rebuild their school lives after the interruption of school activities during the pandemic years. Supportive measures should be implemented to promote economic recovery and reduce unemployment [52–53].

The present findings identify a potential service gap for those in the Low-distress class. Despite an overall improvement in psychological distress in the present sample, 5.2% of the Low-distress class transitioned to the High-distress classes after the COVID-19 pandemic. This finding highlights the neglected mental health needs of the mentally healthy, particularly those who are new to the workforce. It would be helpful for universities to offer workshops focusing on workplace adaptations, financial management skills, and communication skills to better equip undergraduates with the skills needed for career development. Companies are encouraged to set achievable, measurable goals that align with the skills and experience of new recruits and arrange experienced colleagues as mentors to provide hands-on guidance. These programs could help young adults, particularly those who missed out on work placements due to the pandemic, make a better transition into the workforce and reduce the likelihood of mental health problems.

### Limitations

This study has several limitations. Firstly, the present sample was recruited via convenience sampling but not random sampling, which may limit the representativeness of the sample and our findings to the general population. Secondly, only around one-third of the respondents who participated in the 2022 survey participated again in the 2023 survey. The high dropout rate might have confounding effects on the present findings. The low proportion of students in the sample might underestimate the effect of financial distress on transition in psychological distress classes, since they might be less likely to suffer from financial worries. Those with higher levels of social distress in 2022 were more likely to drop out of the survey. Despite the small effect size (*d* = 0.11), this attrition bias could still influence the association between social distress and the transition in psychological distress. Future studies could adopt measures such as increased participant engagement and collecting feedback to improve the participants’ retention. Thirdly, this study only included 1-year follow-up data, we could not compare the psychological distress among young people between these two stages and the pre-pandemic and early pandemic periods. Longitudinal studies with extended follow-up periods are needed to evaluate the long-term effects of the pandemic on the temporal trajectories of psychological distress among young people before, during, and after the COVID-19 pandemic [53]. Finally, this study focused on stressors in various domains but did not include protective factors of psychological distress. Future research should examine the effects of protective factors such as social support, coping strategies, and resilience on psychological distress among young people.

## Conclusion

The present study is among the first to use latent transition analysis to examine the transition and stability of psychological distress classes among young adults in Hong Kong during and after the COVID-19 pandemic. It enhances our understanding of the change in young adults’ psychological distress after the pandemic and the factors associated with the transition. Future policy design and intervention practices should take into account the roles of financial and social stressors in any emerging epidemic and its recovery period.

## Electronic supplementary material

Below is the link to the electronic supplementary material.

Supplementary Material 1

## Data Availability

The raw data analyzed in the presentstudy will be available from the first author upon email request without undue reservation to any qualified researcher.
