# Supplementary material for: Change in psychological distress and associated factors among Hong Kong young adults in post-COVID-19 era: a latent transition analysis
Source: Soc Psychiatry Psychiatr Epidemiol. 2025 May 7;60(12):2823–33. doi: 10.1007/s00127-025-02912-5 (PMC12594645; doi:10.1007/s00127-025-02912-5)
Supplement: Supplementary file 1 — Supplementary Material 1 [file 127_2025_2912_MOESM1_ESM.docx]

**Supplementary data**

| Supplemental Table S1. Ten items of psychological distress symptoms (CHQ10) |
| --- |
| Measure items |
| 1. Felt headache |
| 2. Felt palpitate/heart problem |
| 3. Felt discomfort/pressure in chest |
| 4. Felt shakiness/numbness in limbs |
| 5. Had trouble with sleep |
| 6. Been carrying too much burden |
| 7. Been losing confidence in self |
| 8. Felt nervous and tense |
| 9. Felt worried about family/friends |
| 10. Felt life is entirely hopeless |

Supplemental Table S2. Comparisons between completers and dropouts

| Variables | Dropouts | |  | Completers | | *p* | Cohen *d* / OR |
| --- | --- | --- | --- | --- | --- | --- | --- |
|  | *N* | M (SD) /% |  | *N* | M (SD) /% |  |  |
| Gender (female) | 1370 | 72.0% |  | 577 | 72.6% | 0.797 | - |
| Education level (post-secondary or above) | 1337 | 93.0% |  | 568 | 93.7% | 0.583 | - |
| Student status | 1370 | 47.3% |  | 577 | 38.8% | 0.001 | 1.41 |
| Unemployment | 1370 | 1.5% |  | 577 | 2.4% | 0.137 | - |
| Age | 1370 | 25.68 (5.01) |  | 577 | 25.89 (4.42) | 0.380 | - |
| Psychological distress (CHQ10) | 1370 | 11.14 (6.25) |  | 577 | 11.06 (6.01) | 0.789 | - |
| COVID-19 distress | 1370 | 3.38 (1.03) |  | 577 | 3.33 (1.03) | 0.359 | - |
| Financial distress | 1320 | 2.56 (1.20) |  | 565 | 2.51 (1.19) | 0.374 | - |
| Social distress | 1354 | 2.31 (0.90) |  | 575 | 2.22 (0.85) | 0.028 | 0.11 |

Supplemental Table S3. Profiles comparison of the three latent classes of psychological distress under latent class analysis in 2022 and 2023

| Profiles comparison in 2022: | | | | | |
| --- | --- | --- | --- | --- | --- |
| Variables | Low-distress (Class 1) (*N* = 226) 39.17% *N* (%) | Moderate-distress (Class 2) (*N* = 145) 25.13% *N* (%) | High-distress (Class 3) (*N* = 206) 35.70% *N* (%) | *p* | Post Hoc comparison |
| Gender | 172 (76.3%) | 98 (67.4%) | 152 (74.0%) | 0.088 | - |
| Education level | 210 (92.9%) | 136 (93.7%) | 195 (94.7%) | 0.754 | - |
| Student status | 80 (35.2%) | 52 (36.0%) | 92 (44.6%) | 0.144 | - |
| Unemployment | 2 (0.8%) | 2 (1.1%) | 10 (5.0%) | 0.05 | 1 < 3 |
| Variables | Mean (SE) | Mean (SE) | Mean (SE) | *p* | Post Hoc comparison |
| Age | 26.328 (0.306) | 25.538 (0.390) | 25.650 (0.324) | 0.184 | - |
| COVID-19 distress | 2.981 (0.073) | 3.303 (0.086) | 3.725 (0.068) | 0.000 | 1 < 2 < 3 |
| Financial distress | 1.990 (0.068) | 2.588 (0.101) | 3.007 (0.088) | 0.000 | 1 < 2 < 3 |
| Social distress | 1.856 (0.050) | 2.283 (0.068) | 2.563 (0.065) | 0.000 | 1 < 2 < 3 |
| Profiles comparison in 2023: | | | | | |
| Variables | Low-distress (Class 1) (*N* = 245) 42.46% *N* (%) | Moderate-distress (Class 2) (*N* = 183) 31.72% *N* (%) | High-distress (Class 3) (*N* = 149) 25.82% *N* (%) | *p* | Post Hoc comparison |
| Gender | 184 (74.9%) | 123 (67.1%) | 113 (75.6%) | 0.219 | - |
| Education level | 237 (96.8%) | 117 (96.8%) | 141 (94.5%) | 0.588 | - |
| Student status | 77 (31.4%) | 57 (31.2%) | 46 (30.9%) | 0.993 | - |
| Unemployment | 2 (0.8%) | 5 (2.8%) | 4 (2.7%) | 0.203 | - |
| Variables | Mean (SE) | Mean (SE) | Mean (SE) | *p* | Post Hoc comparison |
| Age | 27.287 (0.287) | 26.590 (0.366) | 26.600 (0.376) | 0.206 | - |
| COVID-19 distress | 2.721 (0.077) | 3.158 (0.085) | 3.228 (0.100) | 0.000 | 1 < (2, 3) |
| Financial distress | 2.340 (0.069) | 2.952 (0.091) | 3.272 (0.107) | 0.000 | 1 < 2 < 3 |
| Social distress | 2.016 (0.053) | 2.363 (0.069) | 2.932 (0.078) | 0.000 | 1 < 2 < 3 |
